# Impact of diabetes mellitus on immunity to latent tuberculosis infection

**DOI:** 10.3389/fcdhc.2023.1095467

**Published:** 2023-01-26

**Authors:** Nathella Pavan Kumar, Subash Babu

**Affiliations:** ^1^ ICMR-National Institute for Research in Tuberculosis, Chennai, India; ^2^ International Centre for Excellence in Research, National Institutes of Health, Chennai, India; ^3^ Laboratory of Parasitic Diseases, National Institutes of Allergy and Infectious Diseases, National Institutes of Health, Bethesda, MD, United States

**Keywords:** latent TB infection, type 2 diabetes mellitus, T cells, cytokines, monocytes

## Abstract

Tuberculosis (TB) is an infectious disease that poses a major health threat and is one of the leading causes of death worldwide. Following exposure to *Mycobacterium tuberculosis* (*M.tb*) bacilli, hosts who fail to clear *M.tb* end up in a state of latent tuberculosis infection (LTBI), in which the bacteria are contained but not eliminated. Type 2 diabetes mellitus (DM) is a noncommunicable disease that can weaken host immunity and lead to increased susceptibility to various infectious diseases. Despite numerous studies on the relationship between DM and active TB, data on the association between DM and LTBI remains limited. Immunological data suggest that LTBI in the presence of DM leads to an impaired production of protective cytokines and poly-functional T cell responses, accounting for a potential immunological mechanism that could leads to an increased risk of active TB. This review highlights the salient features of the immunological underpinnings influencing the interaction between TB and DM in humans.

## Introduction

Type 2 diabetes mellitus (DM) is a key risk factor for tuberculosis (TB) and augments the chances of developing active TB disease (1). Around 15% of global TB cases can be attributed to DM comorbidity. Clinically, DM amplifies TB severity and worsens TB treatment (2), while reciprocally TB impedes glycemic control in DM (3). Latent tuberculosis infection (LTBI) is considered to be a state where the individual is infected with *Mycobacterium tuberculosis (M.tb)*, without any signs or symptoms of active TB (4). It was projected that globally, nearly 1.7 billion people were latently infected with *M.tb* in the year 2014 (5). More recently, a meta-analysis reported that based on the interferon-γ release assays (IGRAs) and tuberculin skin test (TST), the current prevalence rate of LTBI was 24.8% and 21.2%, respectively which accounts for one-fourth of the world’s population (6). Although the total reactivation rate of LTBI is around 10% (7, 8), the risk of reactivation is several fold greater in immunosuppressed patients and also patients with DM (9, 10). However, the exact mechanism of the progress of LTBI to active TB in the presence of DM is still unclear. The generally accepted theory is that DM presents with a weakened immune system.

DM is a gradually rising global epidemic, with 382 million global sufferers in 2014 and an anticipated 592 million estimated by the year 2035 (11, 12). In a meta-analysis, it was reported that DM increases the risk of active TB by three-fold, and the majority (95%) of TB patients were from low-and middle-income countries (1). Rising prevalence of DM is also anticipated, which may markedly amplify the public health impact of this double disease burden (13, 14). Thus, both TB and DM are presently amidst the main global public health priorities. The association of DM with increased TB severity is well described (15). It has been reported that DM is associated with an augmented likelihood of positive sputum smear results, cavitation, delayed sputum conversion, and recurrent TB (1, 16). In addition, it has also been reported that increased susceptibility to TB occurs in patients with DM and this has been ascribed to various factors, including direct effects related to hyperglycemia and insulin resistance and indirect effects related to macrophage and lymphocyte function (17). Although many published studies have reported the association between active TB and DM, very little is known about the association of LTBI with DM. In a meta-analysis published in 2017, DM was associated with a small but significant risk for LTBI (9). Another more recent meta-analysis concluded that DM increased the risk for LTBI by 60% (18). Finally, more recent comprehensive longitudinal data indicate that LTBI is associated with increased diabetes incidence (hazard ratio of 1.4) in US Veterans (19). Several studies have shown enhanced susceptibility to TB in animal models of TB-DM co-morbidity (20). Murine studies have also shown increased inflammation and susceptibility to TB in diabetic mice and enhanced production of several T-cell associated cytokines (21). To study TB susceptibility, Martens et al. (22) used streptozotocin-treated C57BL/6 mice that were hyperglycaemic for < 4 weeks (acute) or > 12 weeks (chronic) before low-dose aerosol challenge with *M. tuberculosis* Erdman. Chronic diabetic mice displayed >1 log higher bacterial burden and more inflammation in the lung compared with euglycemic mice indicating a delayed adaptive immune response to TB during chronic DM (22).

## Host immunity to LTBI

The success of *M.tb* is predominantly due to its ability to stay within the host in an asymptomatic state in the form of latency and reactivate after months, years or even decades in only a minority of individuals. An individual’s lifetime risk of reactivation TB from LTBI depends on their age when infected and the occurrence of any other health condition associated with TB progression (23). Since one fourth of the global population is projected to have LTBI, this provides a huge population base from which TB can appear to fuel its global pandemic (1, 13). Elucidating the immunological responses that result in LTBI resistance or continued control or possibly clearance of *M.tb* or protective immunity are very important in determining correlates of risk for TB (13). Published studies have reported that T cells are very important for effective control of *M.tb* by macrophages in granulomas and various T cell subsets act against a broad array of *M.tb* antigens (24). These T cells subsets can be classified as classical MHC-restricted T cells and donor-unrestricted T cells (DURTs) (25). *M.tb* stimulated human CD4^+^ T cells secrete cytokines and chemokines with the help of macrophages (23, 26). In addition to this, CD4^+^ T cell subsets also deliver key helper functions for immune cells involved in LTBI, which involves CD8^+^ T cell and DURT expansion, and antibody production by B cells (27, 28). CD4^+^ T cells and CD8^+^ T cells from individuals with LTBI exhibit broad responsiveness to *M. tb* peptides (29, 30). Innate immune cells, which includes both lymphoid and myeloid, also have a fundamental role in the host response to M.tb (31–33). Recent publications have highlighted the role of a set of innate cells such as the myeloid-derived polymorphonuclear cells (PMNs) and innate lymphoid cells and DURTs, which are involved in the immune responses to *M.tb* (34). The function of other innate cells in LTBI is less clear.

## Host immunity in type 2 diabetes mellitus

DM is a common chronic non-communicable disease, and is prevalent in a huge proportion of adults. DM can be associated with compromised host immunity that consequently increases the rate of various infections including TB (35). Published studies have revealed that various changes occur in glucose and lipid metabolism which in turn lead to alterations from an anti-inflammatory to pro-inflammatory milieu which makes the DM host more susceptible to bacterial infections (36). It has been reported that during DM, insulin resistance due to insulin signaling inhibition results in a series of immune responses that worsen the inflammatory state, which leads to hyperglycemia (37). During DM, there is an impaired innate immune response due to dysfunction of neutrophils and macrophages (38, 39) and also dysfunction in the adaptive immune response (including T cells) (40–42). DM can also affect cellular immunity which in turn worsens insulin resistance and hyperglycemia (42). Several published reports have demonstrated the diabetes-related mechanisms that impair host defence against pathogens and these mechanisms include suppression of cytokine production, defects in phagocytosis, dysfunction of immune cells, and failure to kill microbial agents (35). Findings from these reports demonstrate that the immune system is altered in DM, which involves altered levels of specific cytokines and chemokines, changes in the number and activation state of various immune cell subsets, and increased apoptosis and tissue fibrosis (43). Together, these changes suggest that inflammation participates in the pathogenesis of type 2 diabetes, but how these changes affect the immune response to bystander antigens or newly acquired infections remains unclear. Currently, a better understanding of the mechanisms by which hyperglycemia impairs host defense against pathogens is essential for the development of novel strategies to treat infections in diabetic patients.

## Innate immune responses of LTBI individuals with DM

To elucidate the phenotypic profile of innate immune subsets at homeostasis, Kumar et al. (44) examined the ex-vivo phenotypic profile of dendritic cells (DC) and monocyte subsets in the whole blood of individuals with LTBI with or without DM. LTBI with DM individuals demonstrated significantly diminished frequencies of both myeloid DC and plasmacytoid DC compared with LTBI without DM individuals Hence, the potential to activate the adaptive immune response by initiating antigen presentation in the periphery could potentially be affected in TB-DM co-morbidity. The functional consequences of this altered phenotypic distribution of DC subsets was not reported (44). In addition, in the same study LTBI with DM individuals exhibited significantly diminished frequencies of classical and intermediate monocytes and significantly elevated frequencies of non-classical monocytes in comparison to LTBI without DM individuals (44). The importance of monocyte subsets in *M.tb* infection suggests that functional modifications in these cells in DM patients will contribute to their enhanced susceptibility to TB. Therefore, TB-DM co-morbidity is also characterized by an altered proportion of monocyte subsets, with the potential to influence innate and adaptive immunity to TB (44). Another recent study (45) has characterized innate lymphoid cells (ILCs) using flow cytometry and findings from that study reported that individuals with LTBI and DM had decreased frequencies of ILC2 and ILC3 in comparison to LTBI or DM alone individuals. In the same study, authors have also reported that ILC producing IFNγ was increased whereas IL-13 expression was diminished in LTBI and DM groups, indicating the fact that LTBI and DM are associated with alteration in the ILC compartment (45). A recent study has reported that LTBI individuals with DM showed TB antigen specific diminished frequencies of γδ T cells expressing Type 1 (IFNγ, TNFα) and Type 17 (IL-17F, IL-22) cytokines, cytotoxic markers (Perforin, granzyme B, granulysin), and immune activation (CD69 and PDL-1) markers compared to LTBI group (46). Another recent study from Kathamuthu GR et al., (47) has shown that TB purified protein derivative (PPD) and whole cell lysate (WCL) specific NK and iNKT cells expressing Type 1 (IFNγ, TNFα, and IL-2), Type 17 [(IL-17A, IL-17F and IL-22) cytokines, and cytotoxic markers (perforin and granzyme B) were significantly reduced in LTBI DM individuals compared to LTBI individuals (47). These findings reveal that the innate immune cell compartment, including γδ T cells, NK cells and iNKT cells are compromised in their ability to respond to *M.tb* antigens in DM individuals. In contrast, another study from Kathamuthu GR et al., (48) on Mucosal-associated invariant T (MAIT) demonstrated that the percentage of MAIT cells expressing Type 1 and Type 17 cytokines and cytotoxic markers were significantly higher in LTBI-DM individuals in comparison to the LTBI individuals, indicating that not all the innate immune cell subsets are impaired in their ability to mount TB-antigen specific or non-specific immune responses (48). Altogether, the published data reveals that DM comorbidity is associated with altered innate immune markers and possibly related to poor correlates of immune protection and elevated mycobacterial pathogenesis in LTBI individuals.

## Adaptive immune responses of LTBI individuals with DM

Published studies have described that immunity to TB is mainly dependent on CD4^+^ T cells and more specifically that CD4^+^ Th1 and Th17 cells play an important role in protective immunity, in both human and animal models (49). Kumar et al. (50) have reported that LTBI with DM individuals exhibited diminished frequencies of mono and dual functional Th1 (IFNγ, IL-2 and TNFα), Th2 (IL-4, IL-5 and IL-13) and Th17 (IL-17A, IL-17F and IFNγ) CD4^+^ T cells at baseline and following TB antigen stimulation in comparison to LTBI without DM individuals, indicating that coincident DM alters the function of CD4^+^ T cells. Further in this article, the authors have also demonstrated that this altered frequency of multifunctional T cells is mainly dependent on IL-10 and TGFβ, since neutralization of either cytokine resulted in significantly augmented frequencies of Th1 and Th2 cells in LTBI with DM individuals (50). Subsequently, the same group (51) also reported that CD8^+^ T cells play an important role in the immunity to LTBI with DM individuals. They reported that LTBI with DM individuals showed diminished frequencies of CD8^+^ T cells producing Tc1 (IFNγ, IL-2 and TNFα), Tc2 (IL-4, IL-5 and IL-13) and Tc17 (IL-17A, IL-17F and IFNγ) cytokines at baseline and upon TB antigen stimulation in comparison to only LTBI individuals. In contrast, CD8^+^ T cells expressing the Granzyme B and perforin were significantly elevated in LTBI with DM group upon TB antigen stimulation, indicating that coincident DM modulate the cytotoxic T cell function in LTBI individuals (51).

Few other published studies (44) have characterized the phenotypic profile of T and B cell subsets, such as memory T cells and memory B cells at homeostasis. Kumar et al. have reported that LTBI with DM individuals demonstrated significantly diminished frequencies of CD4^+^ effector memory T cells in comparison the LTBI individuals (44) Moreover, LTBI with DM individuals showed significantly elevated frequencies of activated memory B cells and atypical B cells and lower frequencies of naïve B cells compared to LTBI, indicating that TB-DM profoundly modulates cells of the adaptive immune system (44). Thus, DM in LTBI appears to be associated with major impairment in T cell activation and function. Thus, functional and phenotypic alterations in the adaptive immune cell compartment in LTBI offer important insights into the potential mechanism by which DM could influence the progression from LTBI to active tuberculosis.

## Immune biomarkers to LTBI individuals with DM

Cytokines play an important role in the host immune response against *M.tb* (52). IFN-γ and TNF-α are critically important for protective immunity in both human infections and animal models (49, 52). Other cytokines such as IL-17A, the prototypical type 17 cytokine, are known to play an essential role in mediating memory immune responses to *M.tb* in mice (53). Another proinflammatory cytokine, IL-22 has been to shown to contribute to the human antimycobacterial response (54). IL-1 family of cytokines, mainly IL-1α and IL-1β, are essential for resistance to tuberculosis (55, 56). In addition, IL-18 (57) and IL-12 (58, 59) are both recognized to be vital in immunity to *M.tb* and finally, IL-6 has also been shown to facilitate inhibition of disease progression (60). A study published by Kumar et al. (61) has described that LTBI with DM individuals displayed diminished circulating levels of type 1 (IFNγ, IL-2 and TNFα), and type 17 (IL-17A, IL-17F and IL-22) cytokines and also other pro-inflammatory cytokines such as IL-1β and IL-18 in comparison to non-DM group. In addition, diminished levels of Type 1 and Type 17 cytokines were seen upon TB antigen stimulation, indicating that presence of DM, is characterized by lower production of protective cytokines, allowing for a probable immunological mechanism that could account for the augmented risk of active tuberculosis in latently infected individuals with DM (61).

Subsequently by Kumar at al., (62) in another study reported that IL-20 subfamily of cytokines are associated with LTBI-DM comorbidity. They studied the association of IL-20 subfamily of cytokines in LTBI-DM co-morbidity because the IL-20 subfamily of cytokines plays an essential role in both host defense mechanisms and glucose metabolism. Findings from this study revealed that individuals with LTBI with DM exhibited diminished systemic plasma levels of IL-10, IL-19, IL-20 and IL-24 but increased levels of IL-22 in comparison to the LTBI group, signifying that the diminished production of cytokines may affect immunity against TB infection (62). In another study, the authors illustrate the effect of coexistent DM on adipocytokine levels in LTBI individuals (63). Findings from this study revealed that LTBI with DM individuals displayed diminished plasma levels of adiponectin and adipsin and heightened plasma levels of leptin, visfatin and PAI-1. Moreover, adiponectin and adipsin revealed a significant negative association, whereas leptin, visfatin and PAI-1 and displayed a significant positive association with HbA1C levels. These findings clearly reveal that due to metabolic dysfunction, an imbalance in the appearance of pro- and anti- inflammatory adipocytokines arises in TB-DM, which is an important feature that could influence the development of TB pathogenesis (63). There was a study by Aravindhan V et al., who have shown that inflammatory markers such as IL-1β, IFNγ and adiponectin were significantly elevated in LTBI individuals with newly diagnosed DM (NDM) in comparison to LTBI negative individuals with NDM (64). Subsequently from the same group, another study demonstrated that there was elevated IL-27 and IL-10 levels in the LTBI NDM, compared to LTBI group (65). Finally, they also reported that IL-38 was significantly reduced in LTBI infected DM in comparison to LTBI negative DM, thus implying that these cytokines play a role in the LTBI-DM nexus (66).

There is a rising body of evidence that indicates that immunocompromise is an important part of DM and that this compromise could have severe consequences in the face of intracellular pathogens like *M.tb (* 61 *).* Thus, DM appears to profoundly alter a variety of host immune biomarkers important in the immune response to *M.tb* in LTBI individuals.

## Effect of pre-diabetes in LTBI individuals

Pre-diabetes (PDM) or intermediate hyperglycemia is an abnormal risk state for DM that is diagnosed if the glucose levels are above the normal thresholds but below the levels of overt diabetes (67). There is an increase in the incidence of PDM globally, and it is projected that over 470 million people will have PDM by 2030 (68). The two core functional defects in PDM are insulin resistance and pancreatic beta-cell dysfunction, and these changes manifest before the occurrence of glucose level abnormalities (67, 69). A relationship of PDM with TB risk is not completely well characterized (70). To characterize the impact of PDM in LTBI, Kumar et al. (71) examined the CD4^+^ and CD8^+^ T cell expression of cytokines in LTBI with coincident PDM. The study findings demonstrated that LTBI-PDM individuals are associated with diminished multifunctional frequencies of CD4^+^ Th1 cells expressing IFNγ, IL-2 and TNFα and Th17 cells expressing IL-17A, IL-17F and IFNγ at baseline and upon TB antigen stimulation in comparison to LTBI only individuals. In addition, the study findings also revealed that LTBI-PDM is also characterized by diminished frequencies of mono-functional CD8^+^ Tc1 (IFNγ, IL-2 and TNFα) and Tc17 (IL-17A, IL-17F and INFγ) cells at baseline and upon TB antigen stimulation, indicating that PDM is correlated with alterations of the immune response in LTBI with compromised CD4^+^ and CD8^+^ T cell function. These study results also imply that diminished T cell cytokine production is an important feature of LTBI-PDM and could possibly contribute to the increased risk posed by PDM in the pathogenesis of active TB (71). Finally, one other study examining the circulating plasma levels of a broader panel of pro and anti-inflammatory cytokines in LTBI-PDM individuals has revealed that systemic levels of IFNγ, IL-2, TNFα, and IL-17F were diminished in comparison to LTBI only individuals, indicating that an imbalance in the pro- and anti-inflammatory cytokine milieu is an important feature of LTBI-PDM comorbidity. Researchers have reported that LTBI individuals with PDM presented with a decreased TB antigen specific frequency of γδ T cells expressing Type 1, Type 17 cytokines, cytotoxic markers, and immune activation (CD69 and PDL-1) markers compared to LTBI individuals without PDM (46). Subsequently, another study from Kathamuthu GR et al., (47) has shown that TB purified protein derivative (PPD) and whole cell lysate (WCL) specific NK and iNKT cells expressing IFNγ, TNFα, and IL-2, IL-17A, IL-17F and IL-22 and cytotoxic markers (perforin and granzyme B) were significantly reduced in LTBI-PDM individuals compared to LTBI individuals (47). Finally, there was also another recent finding from the same group on MAIT cells demonstrating that the percentage of MAIT cells expressing Type 1 and Type 17 cytokines and cytotoxic markers were significantly higher in LTBI-PDM individuals in comparison to the LTBI individuals (48). These findings clearly indicate that LTBI-PDM is associated with major perturbations in innate immune cell activation and function and this could potentially have profound effects on the immunity to TB.

## Conclusions

**Figure 1:**
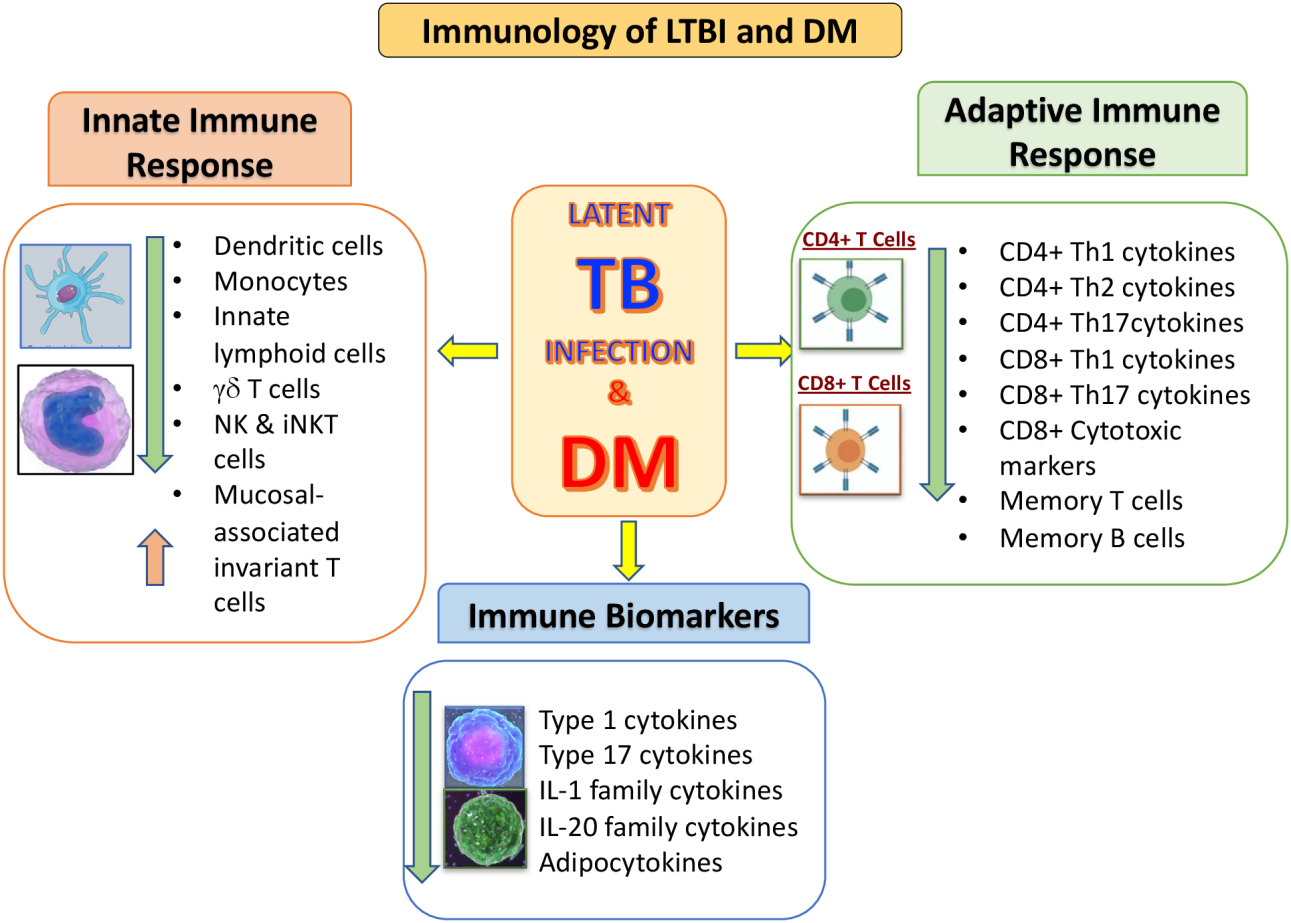
Summary of the immunological interactions between latent tuberculosis infection (LTBI) and type 2 diabetes mellitus **(DM)**. The outcomes of DM on the distinctive arms of the innate and adaptive immune systems is represented by arrows indicating increased or decreased function compared to LTBI without DM.

In conclusion, the mechanisms that are associated with the pathogenesis of TB in the presence of DM are not completely clear. But it is apparent that various immune parameters are altered in the LTBI infected host due to DM, in whom both innate and adaptive immunity is affected (**Figure 1**). Further research is needed for better understanding the biological mechanisms involved, enhance timing of DM testing and clinical care for diabetic or hyperglycemic TB patients. In addition, it would be important to evaluate the potential impact of targeted LTBI screening among the DM individuals.

## Author contributions

NP wrote the first draft of the manuscript. SB contributed to manuscript revision, read and approved the submitted version. All authors contributed to the article and approved the submitted version.
